# Pemphigus Vulgaris with Solitary Toxic Thyroid Nodule

**DOI:** 10.1155/2014/474359

**Published:** 2014-09-21

**Authors:** Mostafa Alfishawy, Karim Anwar, Amira Elbendary, Ahmed Daoud

**Affiliations:** ^1^Department of Medicine, Icahn School of Medicine, Queens General Hospital-Mount Sinai Hospital, New York City, NY 11432, USA; ^2^Department of Internal Medicine, Kasr Al-Ainy University Hospital, Cairo University, Cairo 11559, Egypt; ^3^Dermatology Department, Kasr Al-Ainy University Hospital, Cairo University, Cairo 11559, Egypt; ^4^Ackerman Academy of Dermatopathology, New York City, NY 10016, USA

## Abstract

*Background.* Pemphigus vulgaris is an autoimmune vesiculobullous disease, affecting the skin and mucous membranes. It is reported to be associated with other autoimmune diseases including autoimmune thyroid diseases. However we report herein a case of pemphigus vulgaris associated with autonomous toxic nodule. *Case Presentation.* A 51-year-old woman was evaluated for blisters and erosions that develop on her trunk, face, and extremities, with a five-year history of progressively enlarging neck mass, and a past medical history of pemphigus vulgaris seven years ago. The condition was associated with palpitation, dyspnea, and heat intolerance. Thyroid function tests and thyroid scan were compatible with the diagnosis of thyrotoxicosis due to autonomous toxic nodule. Exacerbation of pemphigus vulgaris was proved by skin biopsy from the patient which revealed histologic picture of pemphigus vulgaris. *Conclusion.* Autoimmune thyroid diseases are reported to associate pemphigus vulgaris. To our knowledge, this case is the first in the English literature to report association between pemphigus vulgaris and autonomous toxic nodule and highlights the possibility of occurrence of pemphigus vulgaris with a nonautoimmune thyroid disease raising the question: is it just a coincidence or is there an explanation for the occurrence of both conditions together?

## 1. Introduction

Pemphigus is a term applied to a group of autoimmune chronic, sometimes fatal, blistering disorders characterized clinically by flaccid blisters that arise on skin and mucous membranes [1]. It is reported to be associated with other autoimmune diseases as myasthenia gravis [2], Sjogren's disease, rheumatoid arthritis [3], and autoimmune diseases of the thyroid [4–7] and accordingly when a pemphigus patient is thyrotoxic, the expected cause would be Graves' disease, which is one of the autoimmune thyroid diseases. Herein we report a case of pemphigus vulgaris associated with thyrotoxicosis due to autonomous toxic nodule.

## 2. Case Presentation

A 51-year-old woman was evaluated for blistering eruption on her face, mouth, trunk, and extremities that develop over one week; they were painful, and she was not able to swallow from the pain. There was no pruritus. She was diagnosed as having pemphigus vulgaris for seven years that was controlled on steroids with a history of occasional exacerbations that required dose adjustment of corticosteroids. Her past medical history included a five-year history of slowly progressively enlarging swelling at front of her neck associated with palpitation, dyspnea, weight loss with increased appetite, nervousness, and heat intolerance. There is no association of diarrhea, polyuria, protrusion of eyes, diplopia, photosensitivity, or blurring of vision. There is no history of fever or trauma, and she denied presence of other swellings in her body.

On physical examination the patient was noted to be irritable. She had swelling in lower part of front of neck, measuring 5 × 8 cm, which was homogenous and firm in consistency. The swelling moves up and down with deglutition. The skin overlying the mass was normal, and the mass was not attached to the skin overlying or surrounding muscles. Both carotids are in place and are felt. No exophthalmos, tremors, or pretibial myxedema noted on examination. On the skin of the face, chest, abdomen, back, and extremities there were flaccid bullae, raw, and denuded erosions with affection of buccal mucosa.

**Figure 1:**
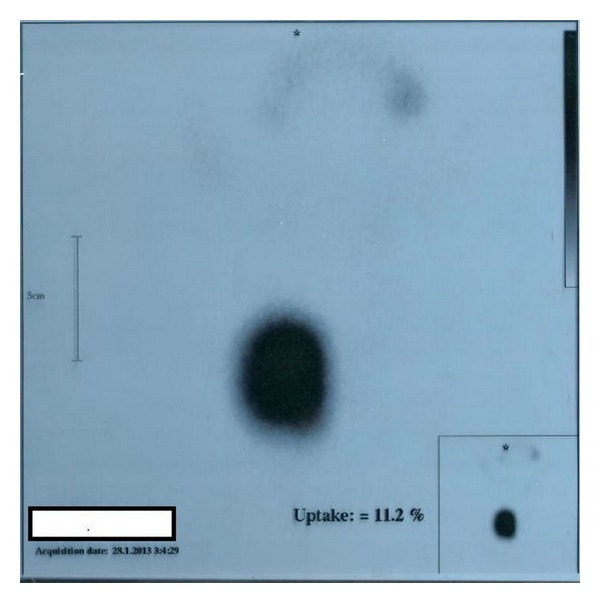
Technetium thyroid scan showing autonomous (hot) nodule, with suppression of uptake in the surrounding tissue.

Laboratory tests including thyroid function tests were done and revealed undetectable level of TSH, with elevated free T_3_ and free T_4_. She was referred for technetium thyroid scan, which demonstrated a homogenous, round focus of relatively increased uptake in the left lobe with suppression of the remainder of the gland (Figure 1), most consistent with autonomous nodule.

Microscopic examination of the skin biopsy obtained from her revealed intraepidermal acantholysis, with split above the basal keratinocytic layer. These findings were compatible with the diagnosis of pemphigus vulgaris in exacerbation.

Patient was informed of different lines of management but she declined surgery or radioactive iodine so treatment was initiated with beta blocker, carbimazole for the thyroid condition, and 60 mg/day prednisone with gradual tapering for pemphigus vulgaris.

## 3. Discussion

Pemphigus vulgaris is an immunobullous disease, where autoantibodies target antigens on cell surface keratinocytes that function in cell to cell adhesion within the epidermis. This is reflected clinically by the flaccid blisters and erosions on the skin and mucous membranes [1].

The association of autoimmune diseases in the same patient has been reported, with a tendency that approaches 25% [8]. Pemphigus vulgaris was reported to be associated with autoimmune diseases including rheumatoid arthritis, Sjogren's syndrome [3], myasthenia gravis [2], and autoimmune thyroid diseases, namely, Graves' disease and Hashimoto's thyroiditis [4–7].

Alteration in thyroid function tests and thyroid autoantibodies were found in pemphigus vulgaris with variable significant results (Table 1).

This predisposition is believed to be genetically determined; Firooz et al. [9] found that first degree relatives of pemphigus vulgaris patients had a threefold increase in the frequency of thyroid disease; in addition, HLA DR3 and HLA D4 were found in high frequency in pemphigus vulgaris and Graves' disease patients [10], emphasizing this theory.

Graves' disease, multinodular goiter, solitary autonomous nodule, and thyroiditis are different causes of hyperthyroidism. Toxic multinodular goiter is presumed to be autoimmune disorder where thyroid stimulating immunoglobulins bind and activate receptors for thyroid stimulating hormone on the thyroid, whereas in case of solitary toxic nodule the role of iodine deficiency and activating mutations in the thyroid stimulating hormone receptor (TSHr) and Gs alpha gene with consequent marked expression of growth factors and their receptors have been implicated [11].

Although previous studies showed the association between autoimmune diseases and so pemphigus vulgaris with autoimmune thyroid diseases, to the best of our knowledge, this is the first report of pemphigus vulgaris to be associated with autonomous toxic nodule. Kahana et al. [12] reported a case of a 44-year-old female patient who presented with pemphigus foliaceus coexisting with toxic nodular goiter. This report raises awareness regarding association between pemphigus vulgaris and a nonautoimmune thyroid disease.

## 4. Conclusion

Pemphigus is an immunobullous disease, reported to be associated with other autoimmune diseases. We reported a case of pemphigus vulgaris and solitary toxic nodule. There is only one previously reported similar case of pemphigus foliaceus and toxic multinodular goiter. To our knowledge, this is the first case to be reported in the English literature and it highlights the possibility of occurrence of pemphigus vulgaris with a nonautoimmune thyroid disease, although it seems most probably an incidental finding, but what remains is raising the following question: is there a relation between pemphigus and solitary toxic nodule or is it just a coincidence?

## Figures and Tables

**Table 1:** The alteration in thyroid function tests and thyroid autoantibodies in patients with pemphigus vulgaris.

Reference	Number of patients	Thyroid abnormality (%)	Anti-TPO (%)
Pitoia et al. [4]	15	Goiter, subclinical hypothyroidism (6.7%) Hashimoto's thyroiditis (6.7%)	40%
Ansar et al. [5]	22	None	22.7%
Michailidou et al. [6]	129	Thyroid disease, not specified (2.6%)	Not stated
Daneshpazhooh et al. [7]	75	Autoimmune thyrotoxicosis, Hashimoto (2.7%) Antithyroglobulin (9.3%)	16%
Leshem et al. [13]	110	Autoimmune thyroid disease, not specified (3.6%)	3.6%
Kavala et al. [14]	80	Hashimoto's thyroiditis (9%) Thyroid disease, not specified (16%)	9%

TPO: thyroid peroxidase.
